# Velocity Selective Networks in Human Cortex Reveal Two Functionally Distinct Auditory Motion Systems

**DOI:** 10.1371/journal.pone.0157131

**Published:** 2016-06-13

**Authors:** Jhao-An Meng, Kourosh Saberi, I-Hui Hsieh

**Affiliations:** 1 Institute of Cognitive Neuroscience, National Central University, Jhongli City, 32001, Taiwan; 2 Department of Cognitive Sciences, University of California Irvine, Irvine, CA, 92697–5100, United States of America; Kyoto University, JAPAN

## Abstract

The auditory system encounters motion cues through an acoustic object’s movement or rotation of the listener’s head in a stationary sound field, generating a wide range of naturally occurring velocities from a few to several hundred degrees per second. The angular velocity of moving acoustic objects relative to a listener is typically slow and does not exceed tens of degrees per second, whereas head rotations in a stationary acoustic field may generate fast-changing spatial cues in the order of several hundred degrees per second. We hypothesized that these two types of systems (i.e., encoding slow movements of an object or fast head rotations) may engage functionally distinct substrates in processing spatially dynamic auditory cues, with the latter potentially involved in maintaining perceptual constancy in a stationary field during head rotations and therefore possibly involving corollary-discharge mechanisms in premotor cortex. Using fMRI, we examined cortical response patterns to sound sources moving at a wide range of velocities in 3D virtual auditory space. We found a significant categorical difference between fast and slow moving sounds, with stronger activations in response to higher velocities in the posterior superior temporal regions, the planum temporale, and notably the premotor ventral-rostral (PMVr) area implicated in planning neck and head motor functions.

## 1. Introduction

The auditory system encounters motion cues through the movement of a sound-emitting object or rotation of the listeners head in a stationary sound field, generating a wide range of naturally occurring velocities from a few to several hundred degrees per second [1–3]. Though a number of prior psychophysical [4–8], neurophysiological [9,10], and neuroimaging [11–13] studies have investigated the mechanisms underlying auditory motion detection, only a rudimentary understanding of this system exists with no consensus on models or even existence of a dedicated auditory motion system [14–16]. This is in marked contrast to studies of visual motion that have extensively mapped out neural mechanisms of visual motion perception and developed detailed computational models of motion and velocity detection [17–21].

The visual and auditory spatial systems, however, are fundamentally different and the mechanisms and models developed for vision cannot easily extend to the auditory domain. The human visual system is highly directional with foveal resolving power of a few seconds of arc [22,23], a line of gaze that may vary with respect to head position, and a peripheral field of view that covers most, but not all of the frontal hemifield [24,15]. The auditory spatial system, however, is omnidirectional, has low spatial resolving power of at best 1° [25,26], with ear positions that are in a fixed reference frame relative to head position. During eye saccades or in response to head movements that do not involve target pursuit, the visual input is selectively inhibited [27–29] whereas the auditory system maintains a continuously open spatial channel. The two motion systems likely play complementary but fundamentally different roles in a complex multimodal field.

The angular velocity of moving acoustic objects relative to a listener is typically slow and does not exceed tens of degrees per second, whereas head rotations in a stationary acoustic field may generate fast-changing spatial cues in the order of several hundred degrees per second [3]. We hypothesized that these two types of systems (i.e., encoding slow movements of an object or fast head rotations) may engage functionally distinct substrates in processing spatially dynamic auditory cues, with the latter potentially involved in maintaining perceptual constancy in a stationary field during head rotations and therefore possibly involving corollary-discharge mechanisms in premotor cortex.

The current study was designed to determine if there exist neural centers associated with encoding auditory motion *velocity*, and if yes, whether these centers categorically distinguish between fast and slow motion cues. No prior neuroimaging study has investigated auditory velocity discrimination. We measured cortical response patterns to sound sources moving at a wide range of velocities from 8 to 180°/s in 3D space. Stimuli were virtual-reality recordings of a real-moving loudspeaker attached to a microprocessor controlled arc that rotated in a circular trajectory in an anechoic chamber. This set up provided recordings of real moving sounds filtered through head-related transfer functions (HRTFs) and hence contained the full complement of dynamic cues associated with natural movements of a sound source [30,31,16]. When played back through headphones in the scanner, these sounds were perceptually externalized and reported by our listeners to be more natural than typical headphone-presented sounds. We found a significant categorical differences between fast and slow moving sounds, with the posterior superior temporal regions, the planum temporale, and notably the right premotor cortical areas associated with neck and head motor planning more responsive to higher velocities of movement, consistent with the hypothesized involvement of motor functions in processing high-velocity motion.

## 2. Materials and Methods

### 2.1 Subjects

15 subjects (7 females) ages 20 to 32 (μ = 24.6; σ = 3.67) participated in this study. All but two were right-handed as identified using the Oldfield questionnaire [32]. They reported no history of audiological or neurological disease. Subjects signed informed-consent forms and were paid for their participation. This study was approved by the Institutional Review Boards of the National Central University, and the National Yang-Ming University, Taiwan, and the Institutional Review Board of the University of California, Irvine.

### 2.2 Stimuli and Apparatus

Stimuli were sinusoidally amplitude-modulated (AM) Gaussian noise bursts with a modulation rate of 8 Hz which has been shown to produce low modulation-detection thresholds and strong cortical responses [33,34]. The modulation depth was 50% to avoid silent gaps during stimulus motion. The purpose was to generate a continuously moving auditory “image” without temporal gaps at any point during source movement. To generate 3D stimuli, sounds were presented through a small loudspeaker (4 cm radius) and recorded through miniature microphones inside the open ear canals of a KEMAR manikin (GRAS Sound & Vibration, Holte, Denmark) at a rate of 44.1 kHz via 16 bit A-to-D converters (Creative Sound Blaster Audigy 2ZS). The sounds were therefore filtered through generalized HRTFs. Recording sessions took place in a steel double-walled acoustically isolated chamber (Industrial Acoustics Company, New York, NY; interior dimensions, 2x2x2 m), the surfaces of which were covered with 10.2-cm acoustic foam wedges (Sonex, Seattle, Washington).

The loudspeaker was attached to a microprocessor controlled arc (Arrick Robotics, model MD-2, Tyler, TX) which rotated in a circular trajectory with a radius of 70 cm around the KEMAR’s head on the azimuthal plane (ear level). Stimuli comprised 6 motion velocities of 8, 15, 30, 60, 90, and 180°/s. The fastest-velocity source moved across the -90° to 90° azimuth in 1s, and the slowest in 22.5 s. It is standard practice in studies of auditory motion perception to report velocity as an angular measure, with the reasoning that psychophysical thresholds for motion detection are affected primarily by angular velocity and angular distance (not absolute velocity or distance), i.e., by dynamically changing interaural time and level cues which are largely unaffected by source distance.

To maintain steady velocity throughout the 180° azimuthal region of interest (frontal hemifield), the rotating arc accelerated to a given velocity prior to reaching the point at which the sound source was activated. Recorded stimuli were then segmented into six azimuthal regions: -90° to -60°, -60° to -30°, -30° to 0°, 0° to +30°, +30° to +60°, +60° to +90°, with negative azimuthal values representing locations to the left, positive values to the right, and 0° directly in front of the head. A 1-ms rise/decay ramp was imposed on each segmented stimulus to avoid abrupt transients. This brief rise/decay ramp was sufficient to eliminate clicks at onset and offset of sounds, and short enough as not to significantly affect the perceived distance traversed (i.e., a long rise/decay time may have shortened the perceived movement distance by effectively eliminating the early and late portions of motion, especially for fast velocities). Stimuli were recorded in both the right and left directions of motion. The segmentation procedure therefore created a set of motion stimuli with different azimuthal start and end points, but which always traversed 30°. This 30° arc was traversed in 167 ms for the fastest velocity (180°/s) and 3.75 s for the slowest velocity (8°/s) employed. In addition, a set of spatially stationary (non-moving) sounds (8Hz AM noise at 50% modulation depth) were generated which were used to identify motion-selective regions by contrasting cortical activation patterns in response to stationary and motion stimuli.

Stimuli were presented at a nominal level of 75 dB SL through MRI compatible insert earphones (Sensimetrics, Model S14) which provide high-quality acoustic stimulus delivery while attenuating scanner noise. These insert earphones are small enough to fit within any head coil and were additionally covered with circumaural ear-covers for further attenuation of low-frequency scanner noise. Subjects reported auditory percepts that were externalized at slightly less distant than that at which sounds were presented from the loudspeaker during stimulus recording. This is expected given the use of generalized transfer functions which produce externalized percepts that are not as strong as those expected from individualized HRTFs. Nonetheless, the virtual reality stimuli used here generate significantly more realistic spatial percepts compared to standard lateralization paradigms in which sounds are perceived intracranially.

MR images were obtained in a Siemens 3T (TIM Trio, Siemens, Erlangen, Germany) fitted with a 12-channel RF receiver head coil located at the National Yang-Ming University. Functional images were acquired using a gradient fast echo-planar T2*-sensitive sequence (TR = 2.16 s, TE = 30 ms, flip angle 90°, matrix 64×64, field of view 192×192 mm) with thirty-six axial slices (slice thickness: 3 mm, gap: 0.5 mm). High-resolution anatomical scans were acquired using a T1-sensitive sequence (TR = 2530 ms, TE = 3.03 ms, flip angle = 7°, voxel size: 1.0×1.0×1.0 mm).

### 2.3 Procedure

Participants were instructed to look directly at a fixation point (plus sign) at the center of a projection screen while auditory stimuli were presented. After termination of the auditory stimulus, the fixation point was replaced with a white filled circle which indicated that the subject should identify the direction of perceived motion (left or right) within 3 seconds on an MRI compatible keypad. The white circle then turned green to indicate that the subject’s response was recorded. Head and eye movements were monitored to ensure no movements occurred during trials within a run.

Each subject then completed 8 test runs and 2 control runs. The entire experimental session, including setup time, lasted approximately 80 minutes per subject. For runs 1 to 8, half the trials were “motion” and half were “stationary” trials. Each trial was 15 seconds in duration, which included 12s of sound presentation and a 3s response interval. The 12 seconds of sound on each trial consisted of a number of exact repetitions of the moving sound recording (30° segments) with no silence between repeats. The duration of each repetition was the time to traverse 30° of arc. Thus, at 8°/s, 3 repeats of ~4s were presented over the 12 seconds. At 90°/s, 36 repeats of 333 ms were presented. Each motion sound trial was “paired” with a stationary sound trial within a run. The “paired” stationary sound was selected to be of a duration that matched that of its paired motion trial, and hence presented the same number of times as the motion stimulus within a trial. For example, a 30°/s velocity trial contained 1s motion stimuli that were presented 12 times, and its “paired” stationary trial comprised a 1s duration stationary sounds that were presented 12 times at random locations within the same azimuthal range as that of the motion stimuli. We should note that for the shortest duration stimuli (fastest rate of repeat during 12 s stimulus presentation), the stationary sources changed locations every 167 ms. In spite of this rapid rate of repeat, subjects reported no noticeable change in perceived extensity.

The order of presentation of the motion and stationary trials were fully randomized within a run, with the contingency that “paired” stimuli were not presented back to back. On a given motion trial, one velocity, one 30° azimuthal arc, and one direction of motion (left or right) were randomly selected without replacement from the set of 72 stimuli (6 velocities x 6 azimuthal arcs x 2 directions). The total number of trials per subject per session was 576. Combined across 15 subjects, this guaranteed a minimum of 30 trials per velocity and azimuth. Each run lasted approximately 5 minutes, with 2 minute breaks between runs.

Run 9 was a control condition in which a diotic broadband Gaussian noise was sinusoidally modulated at 8 Hz (50% depth) and presented as an auditory cortex localizer. This run was used only to confirm general activity within auditory cortex consistent with prior work, but was not used to place constraints on analyses. This run lasted approximately 5 minutes. Run 10 was also a control condition that examined the effects of the number of auditory repetitions within a 15-s trial. This run comprised 4 velocities (30, 60, 90 and 180°/s) by 3 repetition numbers (1, 3, and 6 times within a trial), and hence 12 trials within the run. On each trial of this run, a sound was presented at a fixed motion velocity and a fixed number of times. All permutations of velocity by “number of repetitions” were presented within a run but in a random order across subjects. Run duration was approximately 3 minutes.

### 2.4 Data Analysis

We utilized both a standard whole brain group analysis to replicate previous studies and an ROI-based approach to allow us more power in assessing our specific hypothesis. Data preprocessing and analyses were performed using Matlab software with spm8 toolbox (http://www.fil.ion.ucl.ac.uk/spm/software/spm8/). First, motion correction was performed by creating a mean image from all of the volumes in the experiment and then realigning all volumes to that mean image. The images were then smoothed with an isotropic 6 mm full width half maximum (FWHM) Gaussian kernel. Regression analysis was performed with regressors created by the motion conditions representing velocities of 8, 15, 30, 60, 90, and 180°/sec. The two regressors used in the estimation of the model were the following: For slow-motion condition we entered 8, 15, 30°/sec regressors into the model, and for fast-motion condition we entered 60, 90, 180°/sec regressors into the model. A t-statistic was calculated for each voxel and statistical parametric maps (SPMs) were created for each subject. To test specific hypotheses, linear contrasts were also performed and t-statistics were computed at each voxel to identify regions significantly activated in each static condition compared with corresponding motion conditions. Second-level analysis was then performed on the linear contrasts of the parameter estimates from each participant, treating participants as a random effect and voxel-wise t-tests were performed.

## 3. Results

Several groups have reported activation in the posterior parietal and temporal cortices in response to auditory motion [35,36]. In our study, standard GLM univariate statistical analyses were conducted using SPM8 and Automated Anatomical Labeling of Activations in the SPM Toolbox (AAL, www.cyceron.fr/web) to test whether BOLD responses in ROIs that included the right posterior parietal cortex, superior temporal gyrus, and the supramarginal gyrus were selectively responsive to motion velocity. In each case, we filtered out clusters for which the FDR criterion was not reached. All contrasts were based on the same model using corresponding weighting arrays in each contrast.

### 3.1 Moving versus stationary source contrast

**Fig 1:**
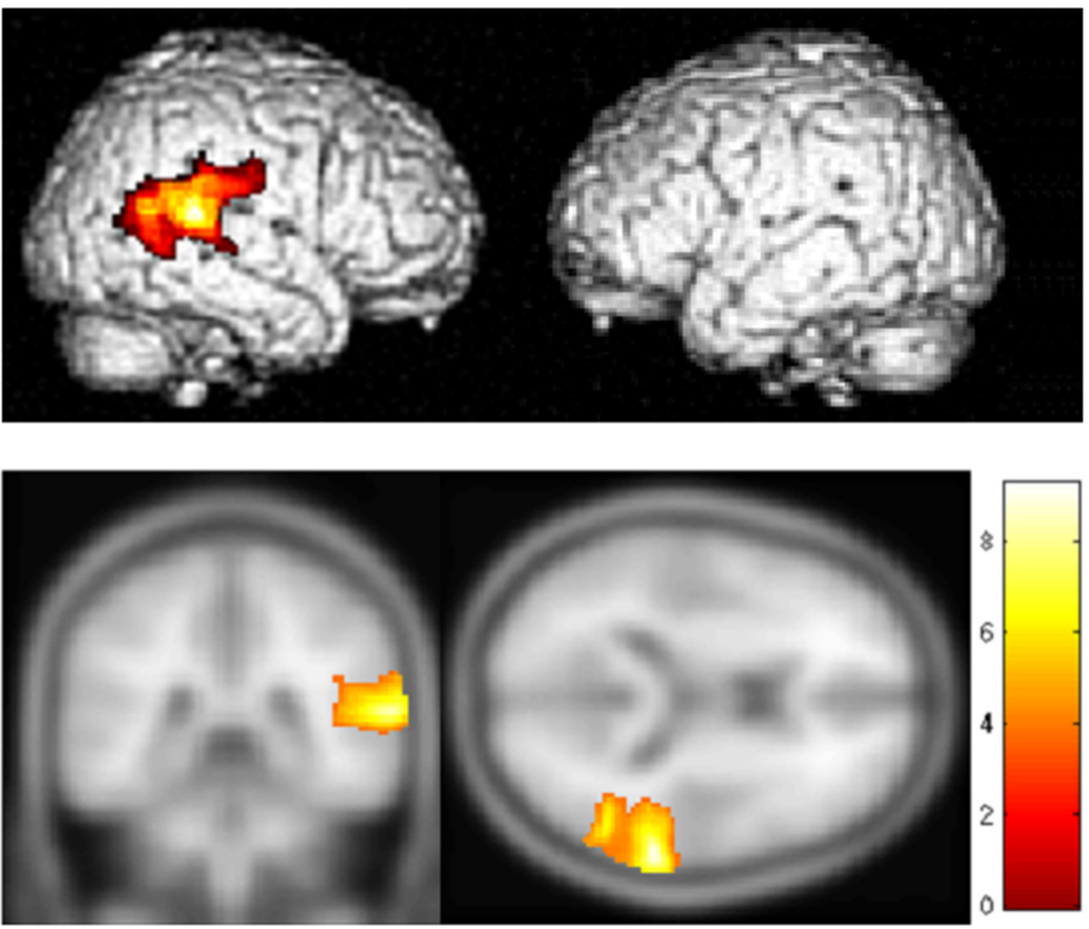
Contrast between motion and stationary source conditions. Group effects for all motion conditions minus stationary conditions as determined by a standard GLM contrast. MNI coordinates for loci of maximum activation are shown in Table 1.

Fig 1 shows group effects from a GLM contrast of all motion conditions minus all stationary conditions. Significant activation (p<0.05, FWE corrected) occurs in the right superior temporal gyrus (rSTG). The activation maps are shown overlaid on SPM8 render and section T1 templates. MNI coordinates of significantly activated areas in the group contrast between motion and stationary conditions are shown in Table 1. This contrast identifies those cortical regions that appear selectively responsive to auditory motion. All significantly activated clusters are in the right hemisphere, with the main peak in the right-STG. This finding is different than those reported by [16] who also used a virtual-space paradigm (i.e., sounds filtered through HRTFs) and found no regions in the auditory cortex that were selective to motion. Other neuroimaging studies, however, have suggested that such motion-selective regions may exist in human cortex [37,38] consistent with identification of motion sensitive neurons in animal models [9]. Smith et al. [16] had also suggested that motion selective regions in human cortex likely do exist, but that prior neuroimaging studies in humans had not appropriately controlled for confounds that potentially resulted in misidentification of motion selective areas.

**Table 1:** Spatial coordinates of local maxima for motion minus stationary contrast.

Coordinate(x, y, z in mm)		Voxel Level (t-score)	Local maxima in cortex labeled in AAL
Left Hemisphere	Right Hemisphere		
	64, -36, 12	9.25	Superior Temporal
	48, -50, 4	7.53	Medial Temporal
	50, -54, 12	6.51	Medial Temporal

Threshold: p<0.001 uncorrected; cluster number > = FDRc.

### 3.2 Fast versus slow movement velocities

**Fig 2:**
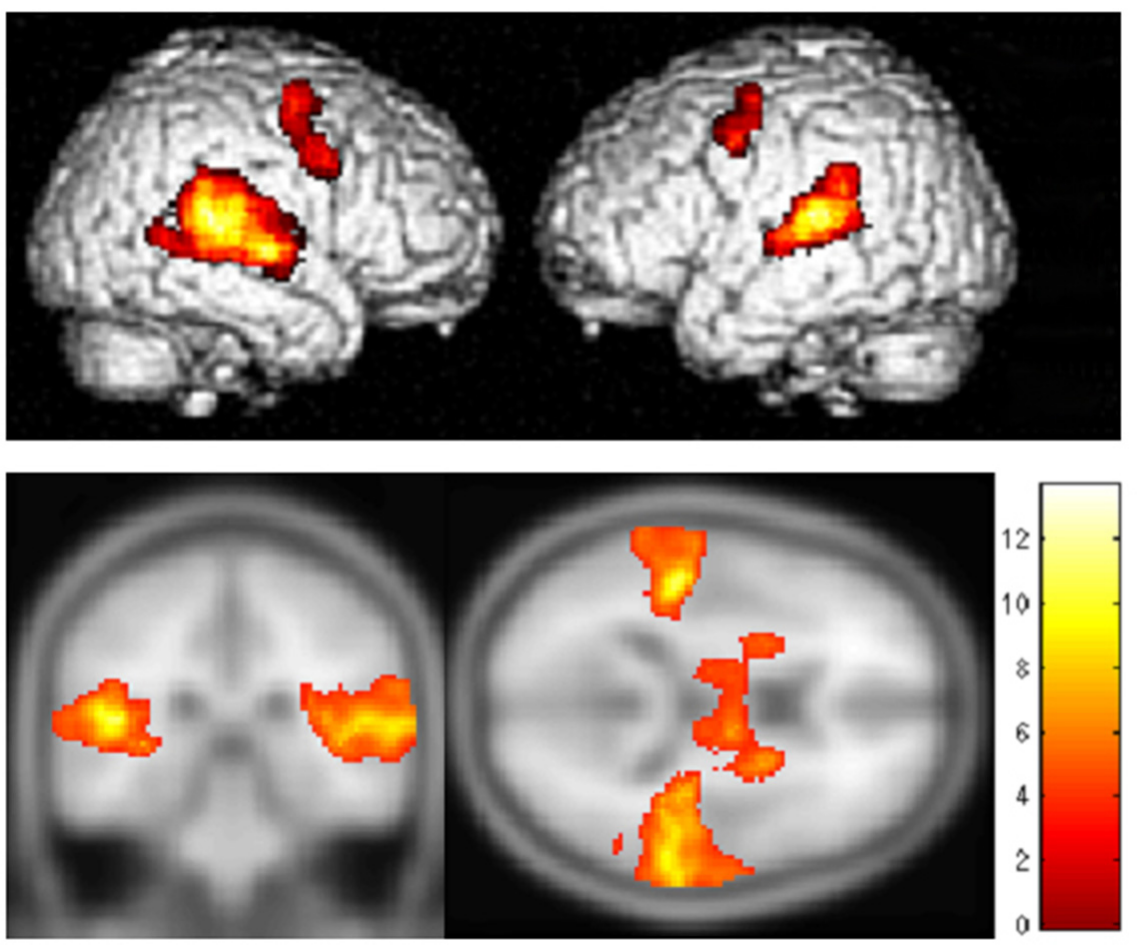
Contrast between fast and slow motion velocities. Group effects for fast (180, 90, 60°/s) minus slow (30, 15, 8°/s) movement velocities as determined from a standard GLM model. MNI coordinates for loci of maximum activation are shown in Table 2.

Standard group GLM contrast was performed between two categories: the three faster motion velocities (180, 90, 60°/s) minus the three slower velocity conditions (30, 15, 8°/s) averaged across all subjects (Fig 2). Analysis of spatially normalized peak data is shown in Table 2, with threshold set at p<0.001 uncorrected, cluster number> = FDRc. Peaks were found bilaterally on the superior temporal cortex, and a number of non-auditory regions including the right precentral gyrus (Broadman area 6), the right precentral sulcus, and the left precentral and middle frontal gyrus. On average, t-values were higher in voxel clusters of the right hemisphere than the left.

**Table 2:** Spatial coordinates of local maxima for fast minus slow velocity contrast.

Coordinate(x, y, z in mm)		Voxel Level (t-score)	Local maxima in cortex labeled in AAL
Left Hemisphere	Right Hemisphere		
	52, -14, 0	13.66	STG (TE 1.0)
	58–34, 12	9.52	pSTG
	52, -4, 54	8.99	PreC/Mid Front G. (6)
	44, 6, 34	8.59	Inf. Front S. (~44)
	38, 0, 58	4.8	Middle Front G.
-48, -30, 14		11.02	STG inside SF (OP 1)
-54, 0, 46		6.94	Precentral G. (Area 6)
-44, -6, 58		4.8	Precentral G. (Area 6)
-40, 0, 40		4.35	Precentral S.

Threshold: p<0.001 uncorrected; cluster number > = FDRc. STG (superior temporal gyrus); SF (sylvian fissure); OP (parietal operculum).

**Fig 3:**
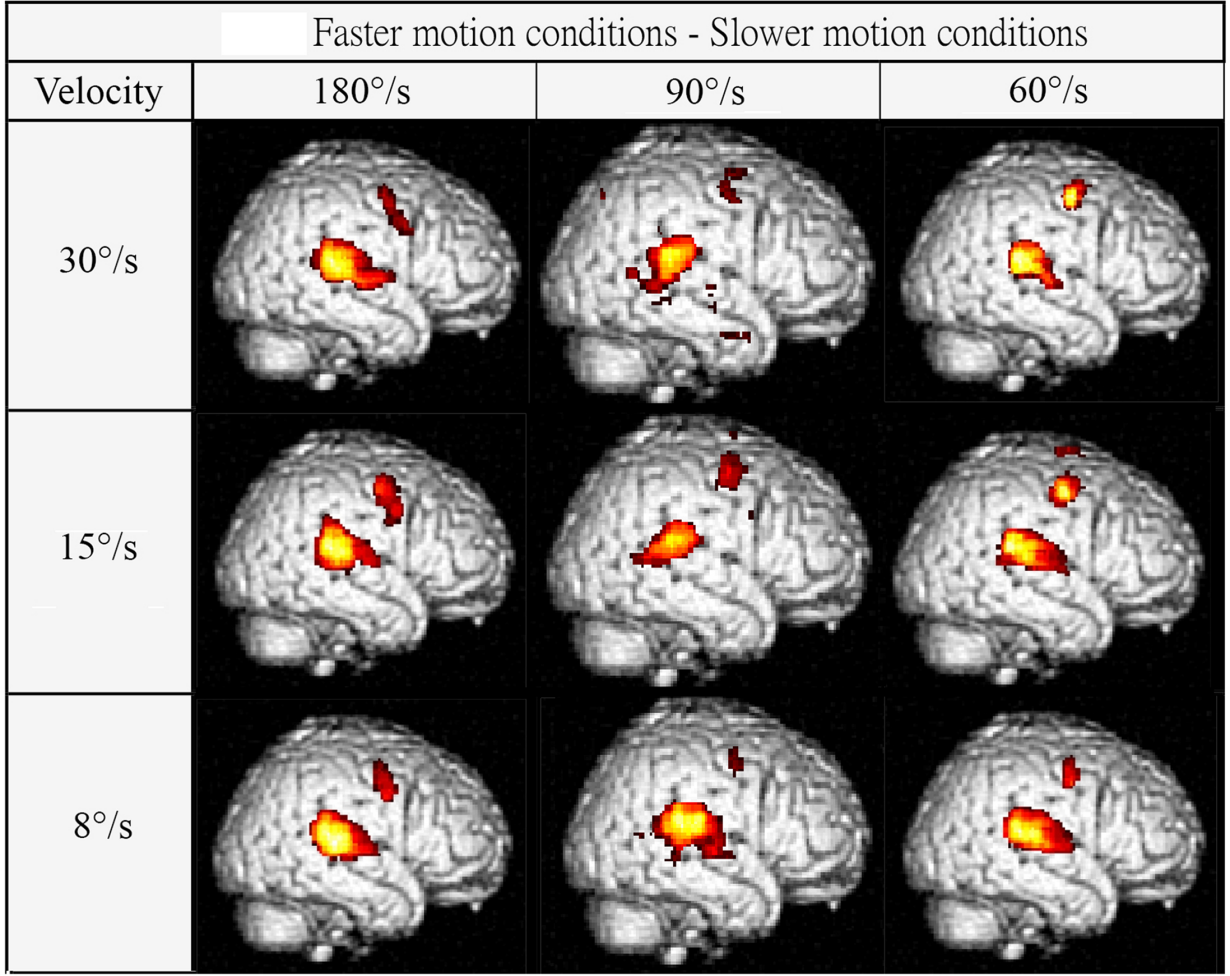
Contrasts between individual fast/slow velocities. Imaging results for group effects comparing individual velocities as determined from a standard GLM model. Each panel shows one fast movement condition (columns) minus one slow movement condition (rows). For example, the top-left panel shows the activation pattern for the 180°/s velocity condition minus the activation associated with the 30°/s velocity condition.

Fig 3 shows group contrasts between individual fast/slow velocities. Each panel shows one fast movement condition (columns) minus one slow movement condition (rows). For example, the top-left panel shows activation patterns resulting from the 180°/s velocity subtracted from activation associated with 30°/s velocity. Pairwise F-tests on activation levels in ROIs across all 6 velocities (15 permutations) are shown in Table 3. Significant activations are only observed between pairs of velocities that are selected across fast (180, 90, 60°/s) and slow (30, 15, 8°/s) velocity categories, but not from within a velocity category. The fastest velocity of motion (180°/s) produces the most significant difference in cortical activation when contrasted to any of the slower motion velocities.

**Table 3:** Pairwise contrasts across velocities.

	180 deg/s	90 deg/s	60 deg/s	30 deg/s	15 deg/s
90 deg/s	n.s.				
60 deg/s	n.s.	n.s.			
30 deg/s	**	**	*		
15 deg/s	**	**	*	n.s.	
8 deg/s	**	*	*	n.s.	n.s.

Threshold: cluster > = number of FDRc

**: p<0.001

*: p<0.05

n.s.: not significant.

Two trends are immediately evident. First, faster velocities when contrasted to slower ones generate stronger activation patterns in STG. Second, and more interestingly, significant bilateral activation is observed in premotor areas (MNI coordinates [52, –4, 54]; [–54, 0, 46]; [–44, –6, 58]) in response to faster velocities of motion. These loci include the premotor ventral-rostral (PMVr) area which has been implicated in planning neck and head motor functions [39,40]. Consistent with this finding, when cortical activation associated with the 3 faster velocity conditions are contrasted to that for stationary sounds, strong activity is observed in premotor areas, whereas when the 3 slower velocities are contrasted to the stationary sound condition, activity in premotor areas are weak and statistically non-significant.

### 3.3 Control for number of stimulus repetitions within a trial

Run 10 was included to ensure that the observed activation patterns are associated with motion velocity and not the number of stimulus repetitions within a 15-s trial. This run comprised 4 velocities by 3 repetition rates. No significant activation differences were observed within a velocity category as a function of the number of stimulus repetitions within a trial. An additional contrast analysis was conducted on the two most extreme *stationary* stimulus conditions that differed in the number of stimulus repetitions within a trial, the stationary condition associated with the 180°/s velocity with 72 repetitions per trial, and the stationary condition associated with the 8°/s velocity with only about 3 repetitions per trial. We observed no activation differences between these two conditions using a Standard group GLM contrast, even at a more liberal threshold of p<0.005 uncorrected, cluster number> = FDRc (see supplementary material at the University of California Data Repository Site). This further confirms that the observed differences in activation patterns between fast and slow velocities do not result from a difference in number of stimulus repetitions within a trial.

## 4. Discussion

Evidence for corollary discharge mechanisms exists in the auditory domain. Prior studies have identified significant bi-directional projections from motor and premotor regions to the auditory cortex [41–43]. Other studies have shown that activity in auditory cortical neurons may be suppressed for several hundred milliseconds *prior* to initiation of motor actions (e.g., vocalization), suggesting an influence of motor-related corollary signals on auditory processing [44,45].

Our findings show that contrasting fast to slow movement velocities generates significant activity in non-auditory premotor regions of cortex associated with planned neck and head motor movements [39,40]. This result is consistent with the hypothesis that processing rapid auditory motion cues, which are produced almost exclusively by normal head rotations, may be categorically distinct from processing slow-velocity movement cues. That head rotation in a stationary sound field generates dynamic auditory motion cues without the percept of movement (i.e., perceptual constancy) suggests that a corollary motor signal must inform the system that the sound source is stationary. A network that uses such a signal may generate activity in motor regions in response to rapidly changing interaural cues that are uniquely associated with head rotation. There is significant evidence that a percept which is normally tightly linked with an action may induce activity in the associated motor regions of the cortex even in the absence of an overt motor output. For example, listening to speech activates motor areas involved in speech production [46] or viewing the motion of human body parts generates activity in premotor regions [47]. Such a system must also be able to induce activity in motion regions of cortex in the absence of explicit motion cues. If the head is rotating in pursuit of a moving sound source, resulting in constant (unchanging) interaural cues, the putative corollary discharge associated with head rotation should generate a percept of motion in spite of constant interaural cues.

While we have discussed here two motion systems (slow vs fast velocity coding), there may in fact exist multiple motion systems in the auditory domain. Two other candidates are the phi-motion system and auditory autokinesis. In phi-motion, when two transient and spatially separated sounds occur within short temporal intervals (<100 ms), a single image is perceived that moves continuously through the spatial extent between the two sources [48–52]. It is noteworthy that the listener has no *a priori* knowledge of the location of the second sound until it has occurred, and therefore the percept of continuous motion must be generated retroactively [51]. Autokinesis is yet another potential auditory motion mechanism likely to have higher-order origins. In autokinesis, stationary auditory objects are perceived as moving even when there has been no prior motion in the sound field [53–55]. This type of movement is extremely slow and occurs both in the free field and through headphone listening, and with or without a visual frame of reference[56]. No neuroimaging or neurophysiological studies have investigated the cortical origins of these motion phenomena.

In summary, we hypothesized the existence of two motion systems with functionally distinct roles in auditory perception. One system codes for naturally moving sound sources with relatively slow velocities (under 30°/s), and the second system codes for rapid velocities resulting from head rotations (often exceeding 100°/s). The first type of system likely involves coding of natural sound sources in motion. These typically generate slow angular velocities. For example, a sound emitting animal or object moving across the azimuth at a distance of 50 m (164 ft) and a velocity of 50 km/h (31mph) would generate an angular velocity of only around 16°/s. A velocity of 50 km/h is a relatively high speed for naturally occurring auditory phenomena, i.e., the type of sounds that would exert adaptive evolutionary pressures. The second type of system, which processes rapid velocities, may involve coordination of concomitant neural signals across sensorimotor and auditory regions, and may potentially be responsible for maintaining perceptual constancy during head movements. We found evidence for this dual system by subtracting cortical activity resulting from slow movement of 3D sounds from that associated with rapid motion, resulting in significant activity in sensorimotor regions of cortex associated with head and neck movement.
